# Integrative Transcriptomic and Perturbagen Analyses Reveal Sex-Specific Molecular Signatures Across Glioma Subtypes

**DOI:** 10.3390/cancers18010052

**Published:** 2025-12-24

**Authors:** Madhu Vishnu Sankar Reddy Rami Reddy, Jacob F. Wood, Jordan Norris, Kathryn Becker, Shawn C. Murphy, Sishir Doddi, Ali Imami, William G. Ryan V, Jennifer Nguyen, Jason Schroeder, Kathryn Eisenmann, Robert E. McCullumsmith

**Affiliations:** 1 Department of Neurosciences and Psychiatry, The University of Toledo College of Medicine, Toledo, OH 43614, USA; madhuvishnusankarreddy.ramireddy@rockets.utoledo.edu (M.V.S.R.R.R.); jacob.wood@rockets.utoledo.edu (J.F.W.); jornorri@umich.edu (J.N.); kathryn.becker@rockets.utoledo.edu (K.B.); shawn.murphy2@rockets.utoledo.edu (S.C.M.); sishir.doddi@rockets.utoledo.edu (S.D.); ali.imami@rockets.utoledo.edu (A.I.); william.ryan2@rockets.utoledo.edu (W.G.R.V.); jennifer.nguyen@rockets.utoledo.edu (J.N.); 2 Division of Neurological Surgery, Department of Surgery, The University of Toledo College of Medicine, Toledo, OH 43614, USA; jason.schroeder5@utoledo.edu; 3 Department of Neurological Surgery, University of California San Diego, San Diego, CA 92093, USA; 4 Department of Cell and Cancer Biology, The University of Toledo College of Medicine, Toledo, OH 43614, USA; kathryn.eisenmann@utoledo.edu; 5 Neuroscience Institute, ProMedica, Toledo, OH 43606, USA

**Keywords:** glioblastoma, GBM, bioinformatics, drug identification, transcriptomics

## Abstract

*Background*: Emerging evidence suggests that biological sex shapes glioma biology and therapeutic response. *Methods*: We performed a sex-stratified analysis of CGGA (Chinese Glioma Genome Atlas) RNA sequencing data comparing low-grade glioma (LGG) with high-grade glioma (HGG) and glioblastoma (GBM). Using the 3PodR framework, we integrated differential expression analysis with Gene Set Enrichment Analysis (GSEA), EnrichR, leading-edge analysis, and iLINCS drug repurposing. *Results*: These comparisons provide a proxy for biological processes underlying malignant transformation. In LGG vs. HGG, 973 significantly differentially expressed genes (DEGs) were identified in females and 1236 in males, with 15.5% and 33.5% unique to each sex, respectively. In LGG vs. GBM, 2011 DEGs were identified in females and 2537 in males, with 12.6% and 30.7% being unique. Gene-level contrasts included GLI1 upregulation in males and downregulation in females, GCGR upregulation in males, MYOD1 upregulation in females, and HIST1H2BH downregulation in males. Additional top DEGs included PRLHR, DGKK, DNMBP-AS1, HOXA9, CTB-1I21.1, RP11-47I22.1, HPSE2, SAA1, DLK1, H19, PLA2G2A, and PI3. In both sexes, LGG–HGG and LGG–GBM grade comparisons converged on neuronal and synaptic programs, with enrichment of glutamatergic receptor genes and postsynaptic modules, including GRIN2B, GRIN2A, GRIN2C, GRIN1, and CHRNA7. In contrast, collateral pathways diverged by sex: females showed downregulation of mitotic and chromosome-segregation programs, whereas males showed reduction of extracellular matrix and immune-interaction pathways. Perturbagen analysis nominated signature-reversing compounds across sexes, including histone deacetylase inhibitors, Aurora kinase inhibitors, microtubule-targeting agents such as vindesine, and multi-kinase inhibitors targeting VEGFR, PDGFR, FLT3, PI3K, and MTOR. *Conclusions*: Glioma grade comparisons reveal a shared neuronal–synaptic program accompanied by sex-specific transcriptional remodeling. These findings support sex-aware therapeutic strategies that pair modulation of neuron–glioma coupling with chromatin- or receptor tyrosine kinase/angiogenic-targeted agents, and they nominate biomarkers such as GLI1, MYOD1, GCGR, PRLHR, and HIST1H2BH for near-term validation.

## Simple Summary

Emerging evidence indicates that biological sex significantly influences glioma biology, progression, and therapeutic response. In this study, we reanalyzed previously published RNA-seq datasets by stratifying glioma samples by sex to identify shared and sex-specific transcriptional programs. Across analyses, both male and female tumors demonstrated consistent dysregulation of neuronal and synaptic signaling pathways, suggesting conserved mechanisms of tumor–neuron interaction. Drug repurposing analyses further highlighted candidate therapeutic classes, including histone deacetylase inhibitors and multi-kinase inhibitors, as potential modulators of these pathways. Collectively, this work expands the growing literature on sex-aware glioma transcriptomics and underscores the value of integrative bioinformatics approaches for informing targeted therapeutic development.

## 1. Introduction

High-grade gliomas (HGGs) are among the most lethal primary brain tumors, comprising World Health Organization (WHO) Grade III astrocytomas and oligodendrogliomas, and Grade IV astrocytomas and glioblastomas (GBMs) [1,2]. GBM, now defined as IDH-wildtype Grade IV astrocytoma, is the most common and aggressive subtype, representing over half of malignant brain tumors with an annual incidence of ~2.7 per 100,000 individuals [1,3,4]. GBM may arise de novo or progress from a lower-grade glioma [5]. The primary therapeutic goals for malignant gliomas are to delay progression, preserve neurologic function, and maintain quality of life. Standard of care includes maximal safe resection followed by concurrent radiotherapy and temozolomide chemotherapy [2,6,7]. In select cases, tumor-treating fields (TTFields), a wearable device that delivers alternating electric fields to disrupt mitosis, inhibits DNA replication, interferes with cell motility, and enhances systemic antitumor immunity, are employed as adjunctive therapy [8]. Despite multimodal treatment, outcomes remain poor, with a median survival for GBM of less than one year and a five-year survival rate of only 6.7% across all ages [4]. These sobering statistics underscore the need to clarify the molecular drivers of glioma biology and identify new therapeutic targets.

Molecular diagnostics now inform glioma classification, prognosis, and treatment selection. Established biomarkers include epidermal growth factor receptor (EGFR) amplification, O-6-methylguanine-DNA methyltransferase (MGMT) promoter methylation, and mutations in isocitrate dehydrogenase 1/2 (IDH1/2). MGMT promoter methylation predicts improved response to alkylating agents such as temozolomide, while IDH mutations correlate with longer survival [9,10,11]. Conversely, EGFR amplification is associated with poorer outcomes [12,13,14]. Although sex-related variability in these molecular alterations has been reported, comprehensive transcriptomic analyses addressing these differences remain limited.

Emerging evidence indicates that biological sex influences glioma incidence, treatment response, and survival. GBM is more common in males, who represent nearly 60% of cases, whereas females often experience longer survival and greater responsiveness to therapy [4,15]. Proposed mechanisms for this disparity include sex-specific differences in hormone signaling, immune modulation, and gene expression, though recent work points to intrinsic, cell-autonomous programs as key drivers. For instance, sex-dependent regulation of tumor suppressor pathways, RB and TP53, enhances astrocyte transformation and tumorigenesis in males in mesenchymal GBM models [16]. Imaging and transcriptomic studies further suggest that necrosis and cell-death patterns may be governed by distinct transcriptional expression profiles, such as MYC in females and TP53 in males [17]. Collectively, these findings support the view that intrinsic, sex-specific molecular programs—beyond hormonal differences—shape glioma biology and progression.

Advances in RNA sequencing (RNA-seq) have enabled systematic characterization of gene- and pathway-level changes in central nervous system tumors [18,19]. Large-scale datasets such as the Chinese Glioma Genome Atlas (CGGA) provide extensive molecular and clinical annotation, allowing stratified analyses by tumor grade, mutation status, and patient sex [20,21]. While prior studies have explored glioma progression across grades or examined sex differences within individual grades, few have comprehensively assessed sex-specific transcriptomic programs associated with grade comparisons [22,23].

In this study, we leveraged RNA-seq data from the CGGA to compare transcriptomic profiles across glioma grades in male and female cohorts. Comparisons between low-grade glioma (LGG) and HGG/GBM provide a proxy for biological processes relevant to malignant transformation. We deployed Gene Set Enrichment Analysis (GSEA) and EnrichR to identify differentially enriched pathways, and used the iLINCS perturbagen database to identify drug repurposing candidates predicted to reverse grade-associated transcriptomic signatures [24]. This integrative framework defines sex-specific molecular features linked to grade comparisons and highlights potential sex-tailored therapeutic strategies.

## 2. Materials and Methods

Data Acquisition: We accessed publicly available RNA sequencing (RNA-seq) datasets from the Chinese Glioma Genome Atlas (CGGA) (mRNAseq_693, mRNAseq325) [21,22,25,26,27,28]. These datasets include primary and recurrent low-grade gliomas, primary glioblastomas (pGBM), and secondary glioblastomas (sGBM) arising from lower-grade tumors, as well as recurrent GBMs. Clinical and molecular annotations were obtained from patients treated at multiple neurosurgical centers in China, with diagnoses confirmed by central pathology review according to the 2007/2016 WHO classification. After filtering for complete transcriptomic data and clinical metadata, a total of 1018 samples were retained, spanning WHO Grades II–IV gliomas. Grade 1 tumors were excluded due to limited data availability and lack of grade annotation.

**Figure 1:**
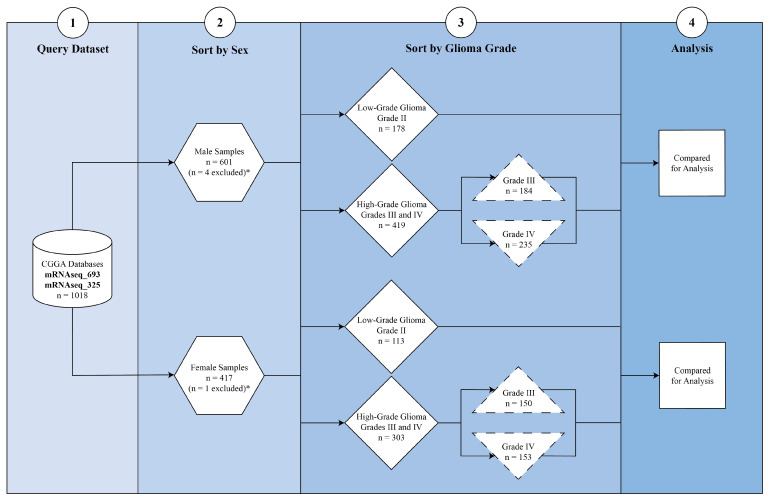
Study cohort selection and stratification workflow. RNA sequencing data were obtained from the Chinese Glioma Genome Atlas (CGGA) (mRNAseq_693 and mRNAseq_325 cohorts), yielding 1018 glioma samples with complete transcriptomic data. Samples were stratified first by sex (male, n = 601; female, n = 417). Four male and one female sample were excluded due to unavailable tumor grade information (*). Remaining cases were sorted by glioma grade: low-grade glioma (LGG, WHO Grade II) and high-grade glioma (HGG, WHO Grade III-IV). Subgroups included male LGG (n = 78), male HGG (n = 419), female LGG (n = 113), and female HGG (n = 303). HGG was further subdivided into Grade III (male n = 184; female n = 150) and Grade IV (male n = 235; female n = 153). These stratified groups were used for sex-specific differential expression and pathway analyses.

Cohort Stratification: Samples were stratified by sex (male vs. female) and histological grade: low-grade glioma (LGG; Grade II), high-grade glioma (HGG; Grades III and IV), and glioblastoma (GBM; Grade IV). Comparison groups were defined as LGG versus HGG and LGG versus GBM within each sex. This stratification enabled the identification of sex-specific molecular features associated with tumor grade (Figure 1). Samples annotated with ambiguous or mixed histologic grades were excluded from differential expression analyses to ensure clear separation between low-grade and high-grade glioma cohorts.

RNA-sequencing and Data Processing: Total RNA was extracted from frozen glioma tissue, with only samples meeting an RNA Integrity Number (RIN) ≥ 6.8 included. Libraries were prepared using the Illumina TruSeq protocol and sequenced on HiSeq 2000/2500/4000 platforms to generate 101–150 bp paired-end reads. Raw reads were aligned to the human reference genome (GENCODE v19, hg19) using STAR (v2.5.2b), and transcript quantification was performed with RSEM (v1.2.31), following the CGGA standard pipeline with batch correction [29,30]. Expressed genes were defined as those with nonzero expression in at least half of the samples. Expression levels were reported as fragments per kilobase of transcript per million mapped reads (FPKM).

Differential Expression Analysis and Visualization: Differential expression analysis was performed using the Limma-Voom R package (v3.5.0) [31,32]. Genes meeting an adjusted *p*-value < 0.05 and |log_2_ fold change| > 1 were defined as differentially expressed genes (DEGs). Each sex-specific comparison (e.g., female LGG vs. female HGG, male LGG vs. male GBM) was analyzed independently to preserve biologically relevant differences. Volcano plots were generated in Python (v3.14.2) using the matplotlib package to visualize statistical gene-level significance and fold-change magnitude [33]. A horizontal dotted line denoted the adjusted *p*-value threshold, while vertical dotted lines marked the log2fold change ± 1. Genes above the horizontal line were considered statistically significant, while those beyond the vertical thresholds were classified as strongly upregulated (right) or strongly downregulated (left).

**Figure 2:**
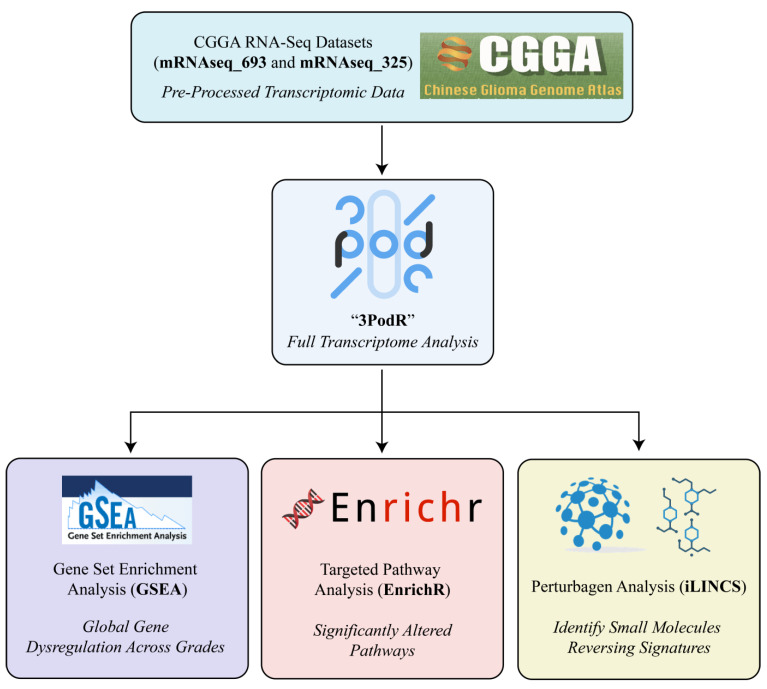
The 3PodR analytic framework for sex-informed drug repurposing. The 3PodR pipeline is a modular, R-based workflow designed to integrate transcriptomic data with drug repurposing analyses. It consists of three sequential analytic pods: (1) GSEA Pod: Gene Set Enrichment Analysis (GSEA) is applied to ranked transcriptomes to identify globally dysregulated pathways between glioma grades in a sex-stratified manner; (2) EnrichR Pod: leading-edge genes from the top GSEA pathways are input into EnrichR for over-representation analysis, enabling focused identification of functionally enriched gene sets using curated databases; (3) iLINCS Pod: refined gene sets are queried in the iLINCS platform to identify small molecules predicted to reverse sex-specific transcriptional signatures. Together, this stepwise framework progresses from broad pathway screening to targeted identification of actionable drug repurposing candidates.

Pathway Enrichment Analysis: Pathway enrichment was performed using Gene Set Enrichment Analysis (GSEA) and EnrichR (Figure 2) [24,34]. GSEA was applied to the full ranked gene lists from each sex-specific comparison to identify pathways significantly enriched in either direction, using curated gene sets [34]. EnrichR was applied to the top 10% of up- and downregulated genes to corroborate GSEA findings and prioritize biologically interpretable pathways [24]. Only pathways significant in both analyses were retained for downstream visualization and interpretation. This dual approach increased confidence in pathway relevance while balancing sensitivity and specificity.

Leading Edge Gene Analysis: To refine pathway findings, leading-edge analysis was performed using GSEA to identify the subset of genes contributing most strongly to each enrichment signal [34]. Three core statistics were evaluated: Tags (proportion of pathway genes contributing to the enrichment score), List (relative ranking of those genes within the dataset), and Signal (a composite metric combining Tags and List). Leading Edge gene analysis prioritizes high-impact genes, highlighting those appearing across multiple enriched pathways within GSEA.

Perturbagen Analysis: Candidate therapeutic compounds were identified using the Library of Integrated Network-based Cellular Signatures (iLINCS) platform (Figure 2) [35]. Gene expression signatures of male and female samples, including LGG vs. HGG and LGG vs. GBM comparisons, were used to query the L1000 small-molecule perturbation dataset. In this framework, concordant signatures represent compounds whose mRNA expression profiles resemble the glioma grade-associated transcriptomic signature, while discordant signatures exhibit an inverse transcriptional pattern. Discordant signatures were prioritized as potential therapeutic candidates because they may reverse disease-associated gene expression states. Compounds were annotated based on available FDA approval status and clinical development stage. Perturbagen predictions generated by iLINCS represent in silico transcriptomic reversal signatures and are intended to be hypothesis-generating rather than evidence of functional or therapeutic efficacy.

Data Integration and Output: All outputs, including differentially expressed genes, enriched pathways, leading-edge genes, and perturbagen predictions, were organized by sex and glioma grade comparison. Results were reported in both tabular and graphical formats, with figures emphasizing high-confidence findings and linking transcriptomic changes to potential therapeutic targets.

## 3. Results

### 3.1. Differentially Expressed Genes (DEGs)

**Figure 3:**
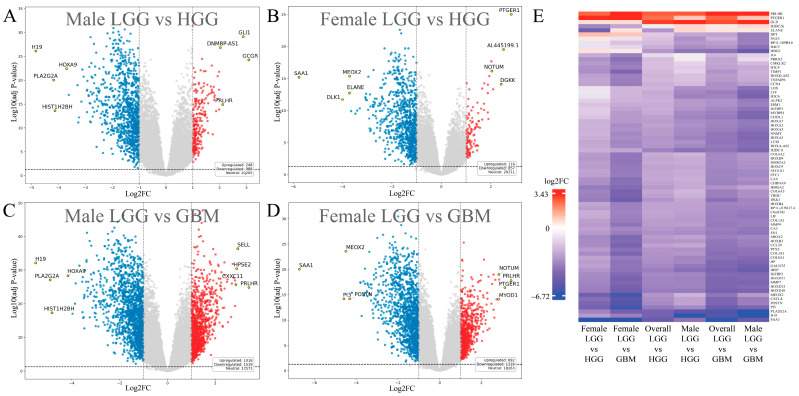
Sex-stratified differential expression analysis in glioma. (**A**–**D**) Volcano plots of differentially expressed genes (DEGs): comparisons of LGG vs. HGG and LGG vs. GBM in males (**A**,**C**) and females (**B**,**D**), with genes of interest and top hits labeled. Red dots represent significantly upregulated genes, and blue dots indicate significantly downregulated genes. (**E**) Hierarchical clustering of representative DEGs: heatmap showing hierarchical clustering of DEG expression patterns across sex-stratified comparisons.

**Figure 4:**
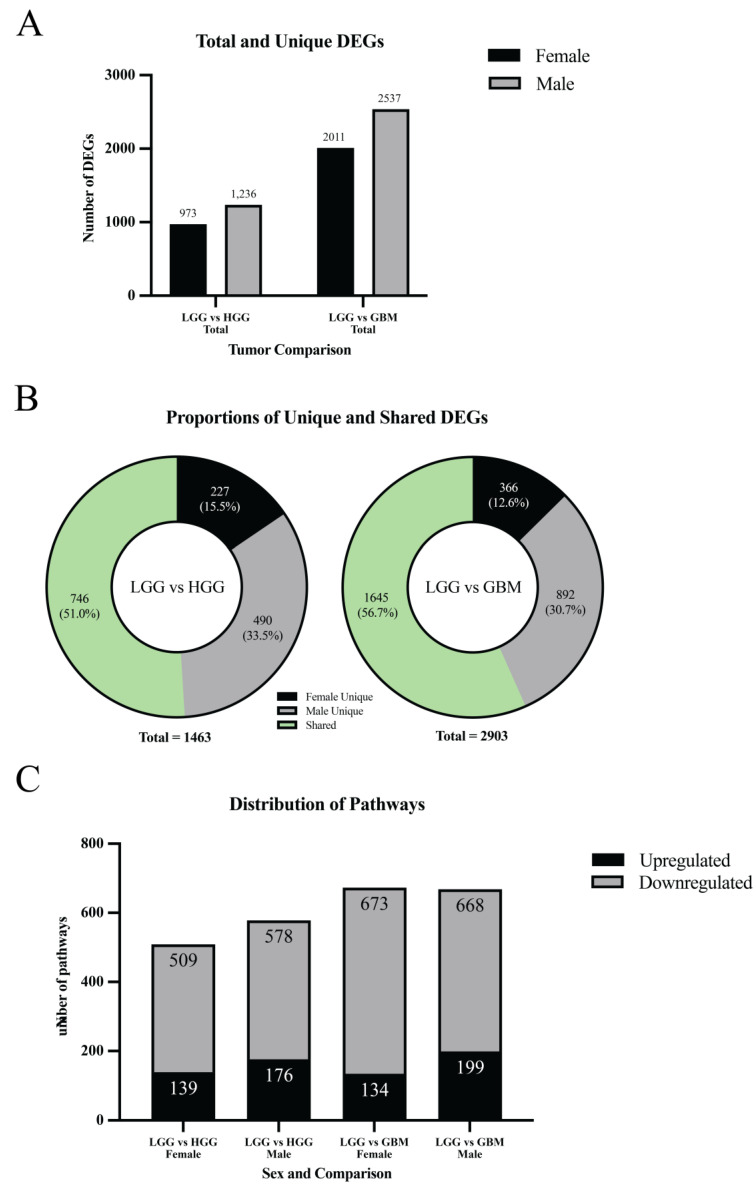
Sex-stratified distribution of DEGs and pathways. (**A**) Total and unique DEGs: bar chart comparing the number of total and sex-specific DEGs across tumor grade comparisons. (**B**) Proportions of unique and shared DEGs: donut plots showing the distribution of male-unique, female-unique, and shared DEGs in LGG vs. HGG and LGG vs. GBM comparisons. (**C**) Distribution of significantly altered pathways: bar plots display the number of upregulated and downregulated pathways in LGG vs. HGG and LGG vs. GBM comparisons for females and males.

*Female LGG* vs. *HGG, Male LGG* vs. *HGG*:

In the LGG vs. HGG comparison, 973 significant DEGs were identified in females and 1236 in males (padj < 0.05, |log_2_FC| > 1) (Figure 3A,B and Figure 4A). Across both sexes, the union contained 1463 distinct DEGs. Of these, 227 were female-unique (15.5%), 490 were male-unique (33.5%), and 746 were shared between sexes (51.0%) (Figure 4B). Directionally, females had 116 upregulated and 857 downregulated DEGs (11.9% up/88.1% down), while males had 248 upregulated and 988 downregulated DEGs (20.1% up/79.9% down). The overall top upregulated genes included *GLI1*, *DGKK*, *GCGR*, *PRLHR*, and *DNMBP-AS1*, while the top downregulated genes were *SAA1*, *DLK1*, *H19*, *PLA2G2A*, and *HOXA9* (Table 1). Detailed male- and female-specific upregulated and downregulated DEG lists are also provided in Table 1. Notably, *GLI1* was strongly upregulated in males, but downregulated in females, and *GCGR* was the most differentially upregulated gene in males, but was significantly downregulated in females. Representative expression patterns of these and other DEGs across comparisons are illustrated in Figure 3E.

*Female LGG* vs. *GBM*, *Male LGG* vs. *GBM*:

In the LGG vs. GBM comparison, 2011 significant DEGs were found in females and 2537 in males (Figure 3C,D and Figure 4A). Across both sexes, the union contained 2903 distinct DEGs. Of these, 366 were female-unique (12.6%), 892 were male-unique (30.7%), and 1645 were shared between sexes (56.7%) (Figure 4B). Directionally, females had 692 upregulated and 1319 downregulated DEGs (34.4% up/65.6% down), whereas males had 1018 upregulated and 1519 downregulated DEGs (40.1% up/59.9% down). The overall top upregulated genes were *PRLHR*, *CTB-1I21.1*, *MYOD1*, *RP11-47I22.1*, and *HPSE2*, while the top downregulated genes were *SAA1*, *PLA2G2A*, *H19*, *HIST1H2BH*, and *PI3* (Table 1). Detailed male- and female-specific upregulated and downregulated DEG lists are also provided in Table 1. *HIST1H2BH* downregulation was specific to males, while *MYOD1* upregulation was specific to females. Representative DEG expression profiles across sex-stratified tumor grade comparisons are shown in the heatmap (Figure 3E). Detailed male- and female-specific upregulated and downregulated DEG lists are provided in Table 1.

### 3.2. Pathway Enrichment Analysis

*Female LGG* vs. *HGG*, *Male LGG* vs. *HGG*:

In the LGG vs. HGG comparison, 509 pathways were significantly altered in females (139 upregulated, 370 downregulated) and 578 in males (176 upregulated, 402 downregulated) (padj ≤ 0.05) (Figure 4C). Both sexes showed a predominance of downregulated pathways, though the proportion of upregulated pathways was slightly higher in males. Representative pathways identified through EnrichR analysis are provided in Supplementary Tables S1–S3, corresponding to female-specific, male-specific, and overall altered pathways, respectively.

*Female LGG* vs. *GBM*, *Male LGG* vs. *GBM*:

In the LGG vs. GBM comparison, 673 pathways were significantly altered in females (134 upregulated, 539 downregulated) and 668 in males (199 upregulated, 469 downregulated) (Figure 4C). Again, both sexes exhibited more downregulated than upregulated pathways, with males showing a greater fraction of upregulation compared to females. Representative pathways identified through EnrichR analysis are provided in Supplementary Tables S1–S3, corresponding to female-specific, male-specific, and overall altered pathways, respectively.

### 3.3. Leading-Edge Gene Analysis

*Female LGG* vs. *HGG*, *Male LGG* vs. *HGG*:

In females, the top upregulated leading-edge genes were *GRIN2B*, *GRIN2A*, *GRIK2*, *GRIN2C*, and *CHRNA7*, with *GRIN2B* contributing to 75 upregulated pathways (Supplementary Table S4). The top downregulated genes were *IL6*, *VEGFA*, *TGFB1*, *ANXA1*, and *TGFB2*, with *IL6* contributing to 208 downregulated pathways. In males, the top upregulated genes were *GRIN2B*, *GRIN2A*, *GRIN1*, *GRIN2C*, and *CHRNA7*, with *GRIN2B* contributing to 105 upregulated pathways (Supplementary Table S5). The top downregulated genes were *IL6*, *VEGFA*, *ANXA1*, *TGFB2*, and *THBS1*, with *IL6* contributing to 221 downregulated pathways (Supplementary Table S6).

*Female LGG* vs. *GBM*, *Male LGG* vs. *GBM*:

In females, the top upregulated leading-edge genes were *GRIN2B*, *GRIN2A*, *GRIN2C*, *GRIN1*, and *CHRNA7*, with *GRIN2B* contributing to the most upregulated pathways (N = 97) (Supplementary Table S4). The top downregulated genes were *TGFB1*, *IL6*, *VEGFA*, *ANXA1*, and *SYK*, with *TGFB1* contributing to 245 downregulated pathways. In males, the top upregulated genes were *GRIN2B*, *GRIN2A*, *GRIN1*, *GRIN2C*, and *CHRNA7*, with *GRIN2B* contributing to 110 upregulated pathways (Supplementary Table S5). The top downregulated genes were *IL6*, *VEGFA*, *ANXA1*, *THBS1*, and *CCL5*, with *IL6* contributing to 214 downregulated pathways.

### 3.4. Perturbagen Analysis

*Female LGG* vs. *HGG*, *Male LGG* vs. *HGG*:

In females, 1456 discordant and 4507 concordant signatures were identified. Top concordant perturbagens included SA-1921085 (0.433), GF-109203 (0.426), and BI-2536 (0.416), while leading discordant perturbagens were *CHEMBL2355554* (−0.377), vindesine (−0.368), and triazolothiadiazine, 36 (−0.359) (Table 2). The most frequent discordant mechanisms of action were VEGFR inhibition, PDGFR tyrosine kinase receptor inhibition, and FLT3 inhibition (Supplementary Table S7). In males, 939 discordant and 3220 concordant signatures were detected. Top concordant perturbagens included SA-1921085 (0.433), GF-109203 (0.426), and BI-2536 (0.416). The strongest discordant agents included IC 261 (−0.335), vindesine (−0.333), and entinostat (−0.332) (Table 3). Discordant mechanisms of action were dominated by PDGFR, VEGFR, and FLT3 inhibition (Supplementary Table S8). Overall results across both sexes are summarized in Table 4, with aggregated mechanisms of action available in Supplementary Table S9.

*Female LGG* vs. *GBM*, *Male LGG* vs. *GBM*:

In females, 809 discordant and 3323 concordant signatures were identified. Leading concordant perturbagens included BI-2536 (0.411), PHA-793887 (0.416), and GF-109203 (0.408), while discordant perturbagens included *CHEMBL2355554* (−0.342), vindesine (−0.337), and IC 261 (−0.333) (Table 2). At the mechanistic level, discordant perturbagens most frequently targeted VEGFR, PDGFR, and FLT3 (Supplementary Table S7). In males, 668 discordant and 3003 concordant signatures were identified. Top concordant perturbagens included BI-2536 (0.416), obatoclax (0.412), and vorinostat (0.428). The most discordant agents were entinostat (−0.332), tacedinaline (−0.327), and parbendazole (−0.345) (Table 3). Discordant mechanisms of action were again enriched for PDGFR, VEGFR, and PI3K inhibition (Supplementary Table S8). Overall LGG vs. GBM results are summarized in Table 4, with full mechanism-level data provided in Supplementary Table S9.

## 4. Discussion

Our integrative transcriptomic analysis reveals robust sex-specific differences in the molecular signatures that accompany glioma biology. By leveraging RNA-seq data from the CGGA, we systematically compared male and female cohorts across LGG vs. HGG and LGG vs. GBM. These two comparisons capture the most biologically and clinically distinct contrasts across glioma grades, providing a practical framework to explore sex-associated molecular differences. This framework is consistent with the clinical reality that HGG and GBM may arise de novo or through stepwise progression from lower-grade gliomas, and thus LGG to HGG/GBM contrasts may illuminate pathways active during malignant transformation [5].

Sex differences emerged at every analytical level. In both comparisons, males exhibited a greater number of unique DEGs than females, with a disproportionate skew toward upregulation. In contrast, female-specific DEGs were fewer and primarily downregulated. This suggests that male gliomas exhibit broader transcriptional activation, whereas female gliomas may involve selective silencing of tumor-suppressive or homeostatic programs. These findings may help explain epidemiologic observations that GBM is more common in males and that females often derive more durable benefit from standard therapies [4,15].

Several genes demonstrated sex-contingent vulnerabilities. GLI1, a Hedgehog effector implicated in glioma proliferation and therapy resistance, was upregulated in males but downregulated in females in LGG vs. HGG [36,37]. GLI1/hedgehog activity is linked to *MGMT* upregulation and reduced temozolomide sensitivity [38]. Hedgehog signaling inhibition resensitizes GBM cells to autophagy in preclinical models [39]. Our findings suggest the hypothesis that hedgehog-directed strategies (or MGMT-modulating combinations) may be therapeutically advantageous in male-predominant contexts, but less so in females, where GLI1 is suppressed. MYOD1 was uniquely upregulated in females in LGG vs. GBM (Table 1), suggesting a potential link to chromatin remodeling and myogenic signaling. Other notable signals included IL6, VEGFA, TGFB1, and ANXA1, genes associated with mesenchymal transition, angiogenic niches, and poor prognosis in glioma [40,41,42]. Their parallel downregulation may reflect grade- and sex- specific remodeling rather than uniform “loss of function,” highlighting the need for spatial transcriptomic or single-cell approaches to resolve whether these shifts represent pathway dampening or redistribution across tumor, vascular, and immune compartments. These sex-stratified transcriptional programs form the central mechanistic framework and primary novelty of the present study.

At the pathway level, a similar divergence was evident. In LGG vs. HGG, 29% of male-specific pathways were upregulated compared to only ~13% in females. In LGG vs. GBM, more than 50% of male-specific pathways were upregulated versus only 3–4% in females (Figure 4). These divergent findings suggest that glioma transcriptomic profiles in males are characterized by widespread activation of oncogenic pathways, while in females, they are characterized by suppression of key regulatory programs. Despite these differences, some pathways were shared. Genes underpinning glutamatergic synaptic signaling (GRIN2B, GRIN2A, GRIN2C, GRIN1, CHRNA7) consistently emerged as leading-edge genes across sexes and grade comparisons, whereas inflammatory and angiogenic programs anchored by IL6, VEGFA, and TGFB1 tended to be suppressed. Collectively, these findings suggest that neuron–glioma synaptic coupling represents a unifying signature of malignant behavior, while collateral pathways may diverge by sex.

Enrichment of glutamatergic synaptic pathways is concordant with prior evidence that glioma cells integrate into neural circuits and receive synaptic input that accelerates tumor growth [43,44]. Neuron-glioma synapses and activity-dependent release of neuroligin-3 are implicated in promoting tumor proliferation and invasiveness [45]. In parallel, pharmacologic interference with NMDA-receptor signaling reduces proliferation, migration, and clonogenic survival in GBM models, consistent with our finding that NMDA/AMPA-related genes may be crucial therapeutic targets for glioma treatment [46,47,48]. Taken together, these data suggest that neuron-glioma coupling is not an epiphenomenon but likely a program that may actively facilitate malignant growth and invasion and represents a rational therapeutic target.

Prior studies have reported sex-based differences in glioblastoma gene expression and molecular architecture. For example, a previous study demonstrated that sex-specific transcriptional programs in adult diffuse gliomas are strongly influenced by IDH mutation status and tumor microenvironmental composition, highlighting the importance of biological context when interpreting sex-associated molecular differences [49]. Similarly, identified sex-dependent gene expression signatures in glioblastoma that implicate pathways related to cell cycle regulation, immune response, and tumor progression [50]. Our findings are consistent with these reports in supporting the presence of sex-associated molecular heterogeneity in glioblastoma, while extending this literature by focusing on pathway-level and systems-oriented analyses that emphasize clinically relevant biological differences rather than isolated gene effects.

We acknowledge that enrichment of synaptic and neuronal gene sets in bulk RNA-seq data may partially reflect neuronal admixture or microenvironmental contamination, particularly in lower-grade or peritumoral samples. Because bulk transcriptomics cannot definitively assign gene expression to specific cell types, single-cell or spatial transcriptomic approaches will be required to resolve cellular origin. Nevertheless, growing evidence of functional neuron–glioma interactions suggests that at least a subset of these signatures may reflect biologically meaningful tumor–neuron crosstalk rather than purely technical contamination.

**Figure 5:**
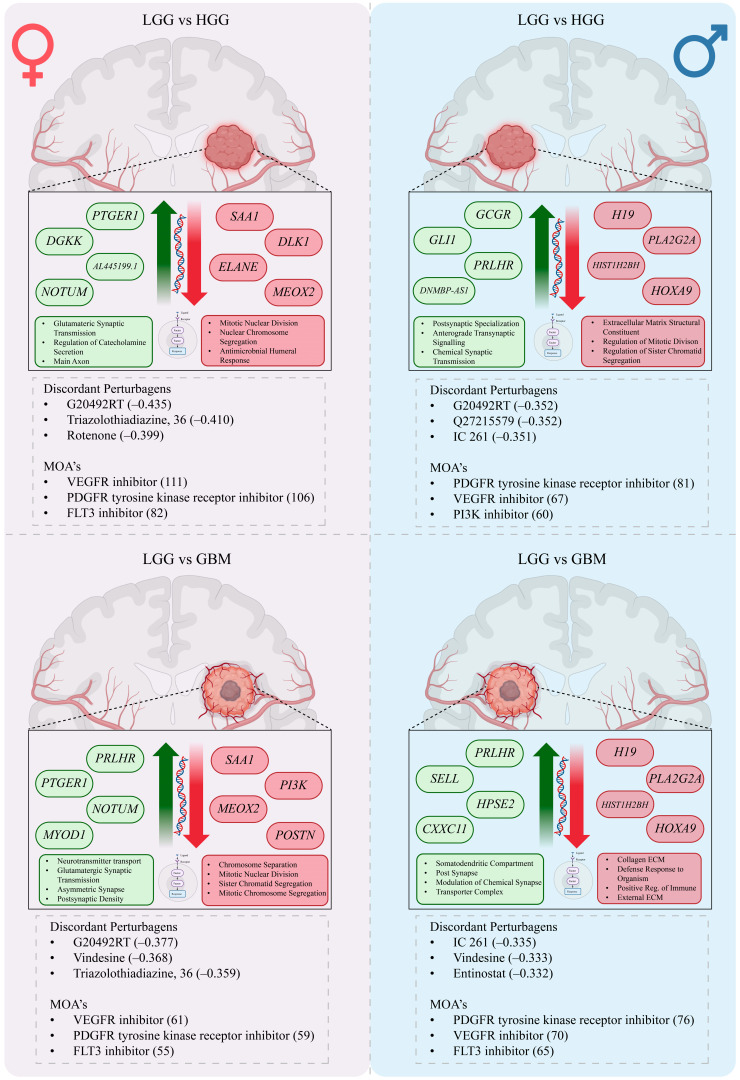
Sex-specific transcriptional programs and perturbagen predictions in glioma. Schematic representation of sex-stratified molecular changes from low-grade glioma (LGG) to high-grade glioma (HGG, top) and glioblastoma (GBM, bottom). Within each comparison, the left hemisphere depicts female-specific changes, and the right hemisphere depicts male-specific changes. Upregulated (green) and downregulated (red) genes and pathways are shown alongside directional arrows. Discordant perturbagens predicted to reverse sex-specific transcriptional signatures are listed below each panel, along with associated mechanisms of action (MOAs). This framework highlights divergent molecular trajectories in male and female gliomas and prioritizes candidate drug classes for sex-informed therapeutic repurposing.

The reciprocal finding, downregulation of cytokine/angiogenic mediators (IL6, VEGFA, TGFB1), should be interpreted in the context of GBM’s profoundly immunosuppressive microenvironment and the limited, often transient, benefit of targeting these processes clinically [51,52]. Aberrant TGF-beta signaling reinforces immune evasion and malignant traits, and VEGF pathway blockade can improve progression-free survival without durable overall-survival gains, in part due to adaptive resistance [53,54]. Our sex-stratified analysis suggests these programs are not uniformly “off” but rather rewired with different emphases by sex (Figure 5), a nuance that may help explain inconsistent clinical responses to single-pathway inhibitors.

Our perturbagen analysis provides a therapeutic context. Among discordant (signature-reversing) agents, recurrent classes emerged: histone deacetylase (HDAC) inhibitors such as entinostat and tacedinaline, tubulin-directed agents including vindesine and benzimidazoles, aurora kinase inhibitors such as tozasertib, and multi-kinase inhibitors targeting VEGFR, PDGFR, FLT3, PI3K, and MTOR, such as OSI 930 (Table 2 and Table 3). Each class has translational support in GBM. Entinostat enhances temozolomide cytotoxicity in models [55,56], while vorinostat is generally well-tolerated and showed modest single-agent activity in recurrent GBM [57]. Tozasertib, a pan aurora kinase inhibitor, induced cell cycle arrest, increased mitochondrial permeability and reactive oxygen species, suppressed growth and migration, and triggered senescence and pro-apoptotic activity in HGG models [58]. Benzimidazoles such as mebendazole display both microtubule-directed and anti-angiogenic activity with strong repurposing potential [59]. OSI 930, which targets KIT and VEGFR, has shown preclinical support in immuno-oncology contexts [60,61]. Importantly, all perturbagen predictions were derived directly from sex-specific differentially expressed gene signatures, ensuring that candidate compounds were nominated based on their ability to reverse male- or female-associated transcriptional programs rather than sex-agnostic profiles.

Two of the iLINCs’ findings merit emphasis. First, vindesine ranks highly among discordant agents for both sexes, yet historical trials in recurrent malignant glioma reported very low response rates [62]. This discrepancy likely reflects historical pathology definitions, patient selection, and the reality that transcriptomic reversal does not guarantee clinical benefit. If revisited, vindesine (or benzimidazole surrogates with better CNS tolerability) should be tested in biomarker-defined and sex-aware designs and in rational combinations (e.g., with synaptic-pathway modulators). Second, dopamine receptor antagonists appeared among concordant agents, meaning they mimicked the grade-associated signature (Figure 5). This is counterintuitive, given evidence that DRD2 blockade impairs GBM stem-like states and synergizes with standard therapy in preclinical systems and early trials [63,64]. We interpret this as an artifact of specific L1000 profiles’ signatures rather than an indication that dopamine antagonism is pro-malignant.

Taken together, our findings suggest sex-aware therapeutic concepts. In females, strong discordance for VEGFR, PDGFR, and FLT3 blockade, coupled with suppression of mitotic-segregation pathways, argues for combining anti-angiogenic or RTK inhibitors with HDAC inhibitors and neuron-glioma coupling modulators (e.g., clinically tractable NMDAR antagonists). In males, where ECM/immune pathways shift and GLI1 rises, hedgehog-directed approaches or chromatin therapies (HDAC/aurora kinase inhibitors) layered onto anti-angiogenic backbones may be more relevant. Across sexes, targeting neuron-glioma synaptic interactions remains a unifying therapeutic axis. Although GLI1 is a well-established marker of glioblastoma malignancy, its expression is highly context-dependent, and the observed downregulation in females may reflect sex-specific modulation of Hedgehog signaling or differences in tumor cellular composition rather than reduced tumor aggressiveness; transcript variant–level effects were not assessed and represent an important limitation of this study.

Of note, a critical limitation in translating transcriptomic perturbagen predictions to glioma therapy is central nervous system (CNS) drug exposure, as many small-molecule inhibitors demonstrate limited or highly variable blood–brain barrier (BBB) penetration. Direct measurements of cerebrospinal fluid (CSF) drug concentrations in CNS tumor patients highlight this heterogeneity, with some targeted agents (e.g., vorinostat, imatinib, ribociclib) achieving measurable CSF levels, while others within the same mechanistic classes remain undetectable despite favorable in silico predictions [65]. These findings underscore that BBB penetration is governed by physicochemical properties, efflux transporter liability, and the unbound drug fraction, rather than the target class alone [65].

This study has other limitations. CGGA cohorts span historical WHO classification schemes, and FPKM-based quantification with inter-center variability may obscure subtle effects. Bulk RNA-seq cannot resolve whether “downregulated” immune or angiogenic genes represent true pathway suppression or redistribution across cellular sub-compartments. Finally, perturbagen predictions identify transcriptomic reversal signatures but do not address drug-specific pharmacokinetics, CNS penetration, or toxicity in vivo. Therefore, validation in sex-matched patient-derived models with functional and electrophysiologic assays is critical to translate these findings for clinical use.

We emphasize that iLINCS-based perturbagen predictions are derived entirely from in silico transcriptomic signature matching and do not constitute experimental validation of therapeutic efficacy. Functional testing in sex-matched glioma cell lines, patient-derived organoids, or in vivo models will be required to determine whether these compounds truly reverse malignant phenotypes or improve treatment response. Nonetheless, systematic perturbagen screening remains valuable because it provides an unbiased framework to prioritize drug classes and mechanisms of action that align with disease-associated transcriptional programs, thereby narrowing the search space for downstream experimental validation.

A key limitation of this study is that all transcriptomic data were derived from the Chinese Glioma Genome Atlas (CGGA), which reflects a Chinese population. Population-specific genetic ancestry, environmental exposures, treatment patterns, and clinical workflows may influence transcriptomic profiles and could limit the generalizability of our findings to non-Chinese or multi-ethnic cohorts. We selected CGGA because it is one of the largest publicly available RNA-seq datasets with complete sex and grade annotation, enabling the stratified analyses required for this work. Nevertheless, future studies should validate the sex-specific transcriptomic programs and perturbagen predictions in independent cohorts such as TCGA and other international datasets to establish the broader applicability of these signatures. Also, the inability to perform differential expression analyses comparing low-grade gliomas with non-GBM high-grade gliomas or astrocytoma-specific subgroups, as the available metadata do not support statistically robust stratification, and such analyses would require a distinct framework beyond the scope of the present work, and a breakdown of glioma subtypes has been classified in Supplementary Table S10. An additional limitation of this study is the lack of detailed tumor location metadata in the CGGA dataset, which precluded adjustment for anatomical tumor site in sex-stratified analyses. As a result, we cannot exclude the possibility that some observed transcriptional differences may be influenced by tumor location in addition to biological sex.

An additional limitation of this study is that hormone-related variables including menopausal status, circulating estrogen or progesterone levels, use of hormonal contraceptives, and hormone replacement therapy were not available in the CGGA dataset. As a result, we were unable to directly disentangle cell-intrinsic sex differences from those potentially influenced by endocrine milieu. Importantly, this limitation reflects the reality of most large, real-world clinical transcriptomic cohorts, where detailed hormonal metadata are rarely captured. Recent transcriptomic analyses of natural menopause show that endocrine aging is accompanied by broad, cell-type–specific transcriptional reprogramming involving differentiation trajectories, stress-response pathways, and developmental signaling networks, rather than isolated hormone-dependent changes [66]. This reinforces the reality that large, real-world transcriptomic cohorts, such as CGGA, capture integrated biological sex effects reflective of clinical populations, even in the absence of granular endocrine metadata

## 5. Conclusions

We re-analyzed CGGA RNA sequencing data across glioma grades in male and female cohorts, integrating GSEA, EnrichR, leading-edge analysis, and iLINCS perturbagen screening. Across sexes, neuronal and synaptic programs with glutamatergic receptor enrichment emerged as a shared feature, while collateral pathways diverged: females demonstrated loss of mitotic and chromosome-segregation signals, and males showed reduction of extracellular matrix and immune-related programs. Perturbagen analysis nominated HDAC and aurora kinase inhibitors, microtubule-targeting agents, and inhibitors of VEGFR, PDGFR, FLT3, PI3K, and MTOR as candidate interventions. Collectively, these findings support sex-aware therapeutic strategies that combine modulation of neuron–glioma coupling with chromatin, receptor tyrosine kinase, and angiogenic targets, and they provide biomarkers for near-term validation in experimental models. By linking sex-specific transcriptional processes to candidate perturbagens, this work provides a rational starting point for future mechanistic and preclinical studies rather than definitive therapeutic recommendations.

## Figures and Tables

**Table 1:** Sex-Specific and Shared Significantly Differentially Expressed Genes (DEGs) in Glioma Comparisons. Representative upregulated and downregulated genes significantly altered (adjusted *p* < 0.05, |log_2_FC| > 1) in sex-stratified comparisons of high-grade glioma (HGG) and glioblastoma (GBM) versus low-grade glioma (LGG). Genes with a single asterisk (*) denote unique significant female DEGs; genes with double asterisks (**) denote unique significant male DEGs. (Log2FC) is also annotated next to HGNC Gene ID.

	LGG vs. HGG			LGG vs. GBM		
	Female	Male	Overall	Female	Male	Overall
Downregulated	*SAA1 (−5.762)*	*H19 (−4.847)*	*SAA1 (−3.867)*	*SAA1 (−6.721)*	*H19 (−5.516)*	*SAA1 (−4.728)*
	*DLK1 (−4.003)*	*PLA2G2A (−4.180)*	*DLK1 (−3.725)*	*PI3 (−4.584)*	*PLA2G2A (−4.908)*	*PLA2G2A (−4.393)*
	*ELANE (−3.720)*	*HIST1H2BH (−4.133)*	*H19 (−3.665)*	*MEOX2 (−4.494)*	** HIST1H2BH (−4.829)	*H19 (−4.279)*
	*MEOX2 (−3.711)*	*HOXA9 (−3.699)*	*PLA2G2A (−3.623)*	*POSTN (−4.308)*	** HOXA9 (−4.163)	*HIST1H2BH (−3.990)*
	*PI3 (−3.651)*	*DLK1 (−3.615)*	*HOXA9 (−3.481)*	*IL8 (−4.199)*	*SAA1 (−3.969)*	*PI3 (−3.882)*
	*POSTN (−3.478)*	*IBSP (−3.291)*	*HIST1H2BH (−3.349)*	*DKK1 (−3.797)*	*HBG2 (−3.870)*	*HOXD10 (−3.617)*
	*CXCL6 (−3.404)*	*HIST1H2BJ (−3.264)*	*PI3 (−3.063)*	*HOXD10 (−3.790)*	*IBSP (−3.837)*	*IBSP (−3.561)*
	*IL8 (−3.303)*	*SAA1 (−3.237)*	*HOXD10 (−3.040)*	*PLA2G2A (−3.734)*	*GALNT5 (−3.684)*	*POSTN (−3.474)*
	*HOXA9 (−3.074)*	*HBG2 (−3.146)*	*IBSP (−3.011)*	*COL6A3 (−3.644)*	** RP11-742B18.1 (−3.565)	*MMP7 (−3.457)*
	*HMGA2 (−3.066)*	*GALNT5 (−3.112)*	*RP11-742B18.1 (−2.955)*	*HOXD13 (−3.629*	*HIST1H3E (−3.554)*	*IL8 (−3.454)*
Upregulated	* PTGER1 (+2.829)	** GCGR (+3.092)	** GLI1 (+2.470)	*PRLHR (+3.418)*	*PRLHR (+3.411)*	*PRLHR (+3.426)*
	*AL445199.1 (+2.515)*	** GLI1 (+2.883)	*DGKK (+2.155)*	*CTB-1I21.1 (+2.846)*	*SELL (+2.946)*	*CTB-1I21.1 (+2.804)*
	*DGKK (+2.419)*	*PRLHR (+2.117)*	** GCGR (+2.096)	*NOTUM (+2.833)*	*HPSE2 (+2.897)*	*MYOD1 (+2.700)*
	*C14orf180 (+2.095)*	*DNMBP-AS1 (+2.029)*	*PRLHR (+2.041)*	* MYOD1 (+2.816)	*CXXC11 (+2.864)*	*HPSE2 (+2.683)*
	* NOTUM (+2.043)	*DGKK (+1.984)*	*DNMBP-AS1 (+1.934)*	*GLP1R (+2.726)*	*F5 (+2.833)*	*AC062021.1 (+2.612)*
	*RP13-439H18.4 (+2.024)*	*SELL (+1.982)*	*F5 (+1.837)*	*RP13-439H18.4 (+2.664)*	*AC062021.1 (+2.832)*	*DNMBP-AS1 (+2.612)*
	*RP11-116O18.1 (+1.941)*	*F5 (+1.979)*	*CCT7P2 (+1.819)*	* PAX2 (+2.617)	*CTB-1I21.1 (+2.779)*	*SELL (+2.600)*
	*PRLHR (+1.906)*	** RP11-266L9.2 (+1.941)	*RP11-116O18.1 (+1.810)*	* C14orf180 (+2.580)	*SFRP2 (+2.698)*	*CXXC11 (+2.563)*
	*GLP1R (+1.878)*	*MYOD1 (+1.873)*	*SELL (+1.790)*	*RP11-116O18.1 (+2.512)*	** GLI1 (+2.668)	*SFRP2 (+2.464)*

**Table 2:** Female-specific perturbagens (iLINCS); Table of the top 10 concordant and discordant perturbagens identified for female LGG vs. HGG and LGG vs. GBM samples.

	LGG vs. HGG		LGG vs. GBM	
	Perturbagen	Score	Perturbagen	Score
Concordant	SA-1921085	0.433	BI-2536	0.411
	GF-109203	0.426	PHA-793887	0.416
	BI-2536	0.416	GF-109203	0.408
	SCHEMBL2557158	0.412	Obatoclax	0.406
	Obatoclax	0.412	PCL2_000057	0.402
	Bithionol	0.410	Bithionol	0.396
	Trichostatin A	0.408	5-Nonyloxytryptamine	0.393
	PCL2_000057	0.408	Brefeldin A	0.392
	Brefeldin A	0.406	SA-1921085	0.420
	Vorinostat	0.428	T 98475	0.437
Discordant	CHEMBL2355554	−0.377	CHEMBL2355554	−0.342
	Vindesine	−0.368	Vindesine	−0.337
	Triazolothiadiazine, 36	−0.359	IC 261	−0.333
	Tozasertib	−0.349	Rotenone	−0.329
	SPECTRUM1505034	−0.346	Triazolothiadiazine, 36	−0.327
	Fenbendazole	−0.345	BRD-K28995283	−0.326
	Rotenone	−0.345	Fenbendazole	−0.326
	IC 261	−0.344	SPECTRUM1505034	−0.326
	OSI-930	−0.343	Tozasertib	−0.326
	SCHEMBL1564574	−0.341	Parbendazole	−0.322

**Table 3:** Male-specific perturbagens (iLINCS); Table of the top 10 concordant and discordant perturbagens identified for male LGG vs. HGG and LGG vs. GBM samples.

	LGG vs. HGG		LGG vs. GBM	
	Perturbagen	Score	Perturbagen	Score
Concordant	SA-1921085	0.433	BI-2536	0.416
	GF-109203	0.426	Obatoclax	0.412
	BI-2536	0.416	Vorinostat	0.428
	SCHEMBL2557158	0.412	PHA-793887	0.433
	Obatoclax	0.412	Brefeldin A	0.429
	Bithionol	0.410	GF-109203	0.466
	Trichostatin A	0.408	BI-2536	0.427
	PCL2_000057	0.408	Bithionol	0.426
	Brefeldin A	0.406	SA-1921085	0.437
	Vorinostat	0.428	T 98475	0.465
Discordant	IC 261	−0.335	Entinostat	−0.332
	Vindesine	−0.333	Tacedinaline	−0.327
	Entinostat	−0.332	Parbendazole	−0.345
	BRD-K28995283	−0.332	Rotenone	−0.345
	CHEMBL2355554	−0.329	6998-75-0	−0.345
	Tacedinaline	−0.327	BRD-K28995283	−0.345
	Parbendazole	−0.324	Vindesine	−0.341
	SCHEMBL1564574	−0.323	OSI-930	−0.341
	CHEMBL2140299	−0.323	IC 261	−0.351
	Tozasertib	−0.321	CHEMBL2355554	−0.352

**Table 4:** Overall perturbagens (iLINCS); Table of the top 10 concordant and discordant perturbagens identified for overall LGG vs. HGG and LGG vs. GBM samples.

	LGG vs. HGG		LGG vs. GBM	
	Perturbagen	Score	Perturbagen	Score
Concordant	T 98475	0.452	BI-2536	0.411
	PHA-793887	0.450	Obatoclax	0.406
	GF-109203	0.433	SA-1921085	0.420
	Brefeldin A	0.429	PHA-793887	0.416
	Cucurbitacin-I	0.428	GF-109203	0.408
	Dovitinib	0.427	PCL2_000057	0.402
	SA-1921085	0.425	Bithionol	0.396
	XMD-1499	0.424	5-Nonyloxytryptamine	0.393
	Palbociclib	0.422	Brefeldin A	0.392
	BI-2536	0.416	T 98475	0.437
Discordant	CHEMBL2355554	−0.371	CHEMBL2355554	−0.342
	Triazolothiadiazine, 36	−0.359	Vindesine	−0.337
	Rotenone	−0.359	IC 261	−0.333
	IC 261	−0.355	Rotenone	−0.329
	Parbendazole	−0.352	Triazolothiadiazine, 36	−0.327
	CHEMBL2140299	−0.350	BRD-K28995283	−0.326
	Vindesine	−0.347	Fenbendazole	−0.326
	SCHEMBL1564574	−0.344	SPECTRUM1505034	−0.326
	6998-75-0	−0.343	Tozasertib	−0.326
	OSI-930	−0.343	Parbendazole	−0.322

## Data Availability

The original contributions presented in this study are included in the article/Supplementary Materials. Further inquiries can be directed to the corresponding author.

## Supplementary Materials

The following supporting information can be downloaded at: https://www.mdpi.com/article/10.3390/cancers18010052/s1, Table S1: Female-specific significantly altered pathways; Table S2: Male-specific significantly altered pathways; Table S3: Overall significantly altered pathways.; Table S4: Female-specific significantly altered leading edge genes (GSEA); Table S5: Male-specific significantly altered leading edge genes (GSEA); Table S6: Overall significantly altered leading edge genes (GSEA); Table S7: Female-specific perturbagen mechanisms of action (iLINCS); Table S8: Male-specific perturbagen mechanisms of action (iLINCS); Table S9: Overall perturbagen mechanisms of action (iLINCS). Table S10: Sample breakdown (sex and tumor grade).

## Abbreviations

**Abbreviations**	**Definition**
CGGA	Chinese Glioma Genome Atlas
DEG	Differentially Expressed Gene
FPKM	Fragments Per Kilobase of transcript per Million mapped reads
GBM	Glioblastoma (WHO Grade IV astrocytoma, IDH-wildtype)
GENCODE	Genome Reference Consortium Gene Annotation
GESA	Gene Set Enrichment Analysis
HGG	High-Grade Glioma (WHO Grades III–IV)
ILINCS	Library of Integrated Network-based Cellular Signatures
LGG	Low-Grade Glioma (WHO Grade II)
PCA	Principal Component Analysis
RNA-seq	RNA Sequencing
TCGA	The Cancer Genome Atlas
TTFields	Tumor-Treating Fields
VEGFR	Vascular Endothelial Growth Factor Receptor
WHO	World Health Organization
