# Supplementary material for: Integrative Transcriptomic and Perturbagen Analyses Reveal Sex-Specific Molecular Signatures Across Glioma Subtypes
Source: Cancers (Basel). 2025 Dec 24;18(1):52. doi: 10.3390/cancers18010052 (PMC12784860; doi:10.3390/cancers18010052)
Supplement: Supplementary file 1 [file cancers-18-00052-s001.zip › cancers-4022023-supplementary.pdf]

	LGG vs HGG	LGG vs GBM
**Downregulated**	Regulation of basement membrane organization	Interleukin-27-mediated signaling pathway
	Platelet-derived growth factor binding	Regulation of basement membrane organization
	Cmg complex	Cysteine-type endopeptidase activity in execution phase of apoptosis
	Insulin-like growth factor II binding	Platelet-derived growth factor binding
	Collagen-containing extracellular matrix	Cmg complex
	Embryonic skeletal system morphogenesis	Insulin-like growth factor II binding
	Double-strand break repair via break-induced replication	Regulation of attachment of spindle microtubules to kinetochore
	Negative regulation of mitotic metaphase/anaphase transition	Collagen-containing extracellular matrix
	Mitotic spindle assembly checkpoint signaling	Negative regulation of mitotic metaphase/anaphase transition
	Mitotic spindle checkpoint signaling	Double-strand break repair via break-induced replication
**Upregulated**	Gamma-aminobutyric acid metabolic process	Clathrin-sculpted gamma-aminobutyric acid transport vesicle
	Regulation of short-term neuronal synaptic plasticity	Clathrin-sculpted gamma-aminobutyric acid transport vesicle membrane
	Synaptic transmission, glutamatergic	Chemical synaptic transmission
	Glutamate catabolic process	Synaptic transmission, glutamatergic
	Regulation of calcium ion-dependent exocytosis	Gamma-aminobutyric acid metabolic process
	Transmitter-gated monoatomic anion channel activity	Glutamate catabolic process
	GABA-gated chloride ion channel activity	GABA-gated chloride ion channel activity
	Ligand-gated monoatomic anion channel activity	Ligand-gated monoatomic anion channel activity
	Synaptic transmission, GABAergic	Synaptic transmission, GABAergic
	Dense core granule	Dense core granule

Supplementary Table S1. Female-specific significantly altered pathways. Female-specific significantly altered pathways identified by EnrichR in LGG vs HGG and LGG vs GBM comparisons.

	LGG vs HGG	LGG vs GBM
**Downregulated**	Cmg complex	Regulation of basement membrane organization
	Platelet-derived growth factor binding	Immunoglobulin receptor binding
	Collagen-containing extracellular matrix	Collagen-containing extracellular matrix
	Collagen fibril organization	Collagen fibril organization
	Double-strand break repair via break-induced replication	Negative regulation of mitotic metaphase/anaphase transition
	Negative regulation of mitotic metaphase/anaphase transition	Mitotic spindle assembly checkpoint signaling
	Mitotic spindle assembly checkpoint signaling	Mitotic spindle checkpoint signaling
	Mitotic spindle checkpoint signaling	Spindle assembly checkpoint signaling
	Spindle assembly checkpoint signaling	Tumor necrosis factor receptor activity
	MHC class II protein complex	MHC class II protein complex
**Upregulated**	Ligand-gated monoatomic anion channel activity	GABA-gated chloride ion channel activity
	Chemical synaptic transmission	Ligand-gated monoatomic anion channel activity
	GABA-gated chloride ion channel activity	Synaptic transmission, GABAergic
	Calcium ion-regulated exocytosis of neurotransmitter	Chemical synaptic transmission
	Synaptic transmission, GABAergic	Benzodiazepine receptor activity
	Regulation of catecholamine secretion	Synaptic transmission, glutamatergic
	Synaptic transmission, glutamatergic	Calcium ion-regulated exocytosis of neurotransmitter
	Transmitter-gated monoatomic anion channel activity	Transmitter-gated monoatomic anion channel activity
	Inhibitory extracellular ligand-gated monoatomic ion channel activity	Gamma-aminobutyric acid signaling pathway
	Regulation of short-term neuronal synaptic plasticity	Neuron projection

Supplementary Table S2. Male-specific significantly altered pathways. Male-specific significantly altered pathways identified by EnrichR in LGG vs HGG and LGG vs GBM comparisons.

	LGG vs HGG	LGG vs GBM
**Downregulated**	Regulation of basement membrane organization	Regulation of basement membrane organization
	Cmg complex	Immunoglobulin receptor binding
	Platelet-derived growth factor binding	Cmg complex
	Collagen-containing extracellular matrix	Platelet-derived growth factor binding
	Collagen fibril organization	Insulin-like growth factor II binding
	Double-strand break repair via break-induced replication	Collagen-containing extracellular matrix
	Negative regulation of mitotic metaphase/anaphase transition	Double-strand break repair via break-induced replication
	Mitotic spindle assembly checkpoint signaling	Negative regulation of mitotic metaphase/anaphase transition
	Mitotic spindle checkpoint signaling	Mitotic spindle assembly checkpoint signaling
	Spindle assembly checkpoint signaling	Mitotic spindle checkpoint signaling
**Upregulated**	Gamma-aminobutyric acid metabolic process	Clathrin-sculpted gamma-aminobutyric acid transport vesicle
	Ligand-gated monoatomic anion channel activity	Clathrin-sculpted gamma-aminobutyric acid transport vesicle membrane
	Chemical synaptic transmission	Chemical synaptic transmission
	GABA-gated chloride ion channel activity	Synaptic transmission, glutamatergic
	Synaptic transmission, GABAergic	Ligand-gated monoatomic anion channel activity
	Regulation of catecholamine secretion	Neuron projection
	Synaptic transmission, glutamatergic	Regulation of short-term neuronal synaptic plasticity
	Transmitter-gated monoatomic anion channel activity	Behavioral fear response
	Neuron projection	Gamma-aminobutyric acid metabolic process
	Inhibitory extracellular ligand-gated monoatomic ion channel activity	Glutamate catabolic process

Supplementary Table S3. Overall significantly altered pathways. Overall significantly altered pathways identified by EnrichR in LGG vs HGG and LGG vs GBM comparisons.

	LGG vs HGG		LGG vs GBM	
	**Gene**	**Pathway Count (N)**	**Gene**	**Pathway Count (N)**
**Downregulated**	*IL6*	208	*TGFB1*	245
	VEGFA	194	*IL6*	238
	TGFB1	183	*VEGFA*	213
	ANXA1	173	*ANXA1*	196
	TGFB2	171	*SYK*	175
	PYCARD	157	*TGFB2*	175
	THBS1	157	*IL1B*	174
	GATA3	154	*THBS1*	174
	CCL5	146	*CCL5*	171
	HLA-DRB1	143	*HLA-DRB1*	161
**Upregulated**	*GRIN2B*	75	*GRIN2B*	97
	GRIN2A	73	*GRIN2A*	94
	GRIK2	71	*GRIN2C*	92
	GRIN2C	70	*GRIN1*	90
	CHRNA7	65	*CHRNA7*	84
	CHRNB2	65	*GRIK2*	84
	NLGN1	65	*CHRNB2*	83
	GRIK4	64	*GRIK4*	76
	GRID2	60	*CHRNA3*	69
	GRIA1	59	*GRID2*	66

Supplementary Table S4. Female-specific significantly altered leading edge genes (GSEA)

	LGG vs HGG		LGG vs GBM	
	**Gene**	**Pathway Count (N)**	**Gene**	**Pathway Count (N)**
**Downregulated**	*IL6*	221	*IL6*	214
	VEGFA	194	*VEGFA*	183
	ANXA1	188	*ANXA1*	180
	TGFB2	169	*THBS1*	159
	THBS1	163	*CCL5*	157
	CCL5	161	*HLA-DRB1*	156
	GATA3	158	*IL1B*	153
	WNT5A	158	*TGFB2*	153
	SHH	153	*WNT5A*	152
	HLA-DRB1	152	*SYK*	150
**Upregulated**	*GRIN2B*	105	*GRIN2B*	110
	GRIN2A	100	*GRIN2A*	103
	GRIN1	98	*GRIN1*	100
	GRIN2C	95	*GRIN2C*	100
	CHRNA7	85	*CHRNA7*	93
	CHRNB2	85	*CHRNB2*	93
	GABRA2	78	*NLGN1*	88
	GRIK2	78	*GRIK2*	82
	DRD4	76	*GABRA2*	79
	GRIK4	73	*KCNB1*	79

Supplementary Table S5. Male-specific significantly altered leading edge genes (GSEA)

	LGG vs HGG		LGG vs GBM	
	**Gene**	**Pathway Count (N)**	**Gene**	**Pathway Count (N)**
**Downregulated**	*IL6*	223	*IL6*	228
	VEGFA	208	*VEGFA*	206
	ANXA1	189	*ANXA1*	191
	TGFB2	176	*THBS1*	172
	THBS1	167	*TGFB2*	168
	GATA3	165	*IL1B*	167
	CCL5	161	*CCL5*	165
	WNT5A	158	*SYK*	163
	SHH	156	*HLA-DRB1*	161
	HLA-DRB1	153	*LYN*	157
**Upregulated**	*GRIN2B*	99	*GRIN2B*	107
	GRIN2A	95	*GRIN2A*	101
	GRIN1	92	*GRIN1*	98
	GRIN2C	90	*GRIN2C*	98
	CHRNA7	82	*CHRNA7*	93
	CHRNB2	82	*CHRNB2*	91
	GRIK2	78	*NLGN1*	86
	GABRA2	75	*GRIK2*	84
	NLGN1	74	*GRIK4*	77
	GRIK4	70	*KCNB1*	75

Supplementary Table S6. Overall significantly altered leading edge genes (GSEA)

	LGG vs HGG		LGG vs GBM	
	**MOA**	**Count**	**MOA**	**Count**
**Concordant**	VEGFR inhibitor	143	Dopamine receptor antagonist	181
	HDAC inhibitor	132	Serotonin receptor antagonist	180
	Dopamine receptor antagonist	128	VEGFR inhibitor	155
	PDGFR tyrosine kinase receptor inhibitor	128	Adrenergic receptor antagonist	142
	FLT3 inhibitor	118	PDGFR tyrosine kinase receptor inhibitor	138
	Serotonin receptor antagonist	117	HDAC inhibitor	133
	CDK inhibitor	116	FLT3 inhibitor	118
	Adrenergic receptor antagonist	111	CDK inhibitor	113
	KIT inhibitor	109	KIT inhibitor	109
	Tubulin inhibitor	94	Dopamine receptor agonist	102
**Discordant**	VEGFR inhibitor	61	VEGFR inhibitor	111
	PDGFR tyrosine kinase receptor inhibitor	59	PDGFR tyrosine kinase receptor inhibitor	106
	FLT3 inhibitor	55	FLT3 inhibitor	82
	PI3K inhibitor	53	KIT inhibitor	74
	Mitochondrial complex I inhibitor	51	PI3K inhibitor	71
	Mitochondrial G3PD inhibitor	51	RET inhibitor	64
	NFkB pathway inhibitor	48	Serotonin receptor antagonist	63
	HDAC inhibitor	47	HDAC inhibitor	62
	CDK inhibitor	46	VEGFR inhibitor (duplicate entry)	59
	MTOR inhibitor	46	Bcr-Abl kinase inhibitor	55

Supplementary Table S7. Female-specific perturbagen mechanisms of action (iLINCS)

	LGG vs HGG		LGG vs GBM	
	**MOA**	**Count**	**MOA**	**Count**
**Concordant**	VEGFR inhibitor	134	Dopamine receptor antagonist	140
	Dopamine receptor antagonist	131	VEGFR inhibitor	135
	HDAC inhibitor	128	HDAC inhibitor	128
	PDGFR tyrosine kinase receptor inhibitor	126	PDGFR tyrosine kinase receptor inhibitor	127
	FLT3 inhibitor	117	Serotonin receptor antagonist	123
	Serotonin receptor antagonist	111	FLT3 inhibitor	118
	KIT inhibitor	109	Adrenergic receptor antagonist	116
	CDK inhibitor	108	CDK inhibitor	111
	Adrenergic receptor antagonist	106	KIT inhibitor	109
	Tubulin inhibitor	80	Tubulin inhibitor	85
**Discordant**	PDGFR tyrosine kinase receptor inhibitor	76	PDGFR tyrosine kinase receptor inhibitor	81
	VEGFR inhibitor	70	VEGFR inhibitor	67
	FLT3 inhibitor	65	PI3K inhibitor	60
	PI3K inhibitor	58	KIT inhibitor	57
	KIT inhibitor	52	FLT3 inhibitor	54
	MTOR inhibitor	51	MTOR inhibitor	51
	HDAC inhibitor	47	HDAC inhibitor	48
	Bcr-Abl kinase inhibitor	41	Bcr-Abl kinase inhibitor	41
	CDK inhibitor	40	PI3K class I inhibitor	35
	Voltage-gated potassium channel blocker	40	Tubulin inhibitor	34

Supplementary Table S8. Male-specific perturbagen mechanisms of action (iLINCS)

	LGG vs HGG		LGG vs GBM	
	**MOA**	**Count**	**MOA**	**Count**
**Concordant**	VEGFR inhibitor	134	Dopamine receptor antagonist	144
	HDAC inhibitor	131	VEGFR inhibitor	136
	PDGFR tyrosine kinase receptor inhibitor	126	PDGFR tyrosine kinase receptor inhibitor	127
	FLT3 inhibitor	117	HDAC inhibitor	125
	Dopamine receptor antagonist	114	Serotonin receptor antagonist	123
	KIT inhibitor	109	FLT3 inhibitor	117
	CDK inhibitor	108	CDK inhibitor	113
	Serotonin receptor antagonist	107	Adrenergic receptor antagonist	112
	Adrenergic receptor antagonist	102	KIT inhibitor	109
	PI3K inhibitor	80	PI3K inhibitor	83
**Discordant**	VEGFR inhibitor	47	PDGFR tyrosine kinase receptor inhibitor	81
	HDAC inhibitor	46	VEGFR inhibitor	67
	MTOR inhibitor	45	PI3K inhibitor	59
	FLT3 inhibitor	44	KIT inhibitor	57
	PI3K inhibitor	44	FLT3 inhibitor	54
	CDK inhibitor	42	MTOR inhibitor	51
	Voltage-gated potassium channel blocker	40	HDAC inhibitor	49
	PDGFR tyrosine kinase receptor inhibitor	33	Bcr-Abl kinase inhibitor	41
	Tubulin inhibitor	32	Tubulin inhibitor	36
	KIT inhibitor	30	CDK inhibitor	33

Supplementary Table S9. Overall perturbagen mechanisms of action (iLINCS)

Dataset	Sex	Total Samples	NA	WHO Grade II	WHO Grade III	WHO Grade IV
mRNAseq_693	Female	295	1	72	120	102
mRNAseq_693	Male	398	0	116	135	147
mRNAseq_325	Female	122	0	41	30	51
mRNAseq_325	Male	203	4	62	49	88

Supplementary Table S10. Sample breakdown (sex and tumor grade)
